# The effects of an IV fluid bolus on mitral annular velocity and the assessment of diastolic function: a prospective non-randomized study

**DOI:** 10.1186/s12871-024-02503-y

**Published:** 2024-03-26

**Authors:** Sebastian Ayala, Orode Badakhsh, David Li, Neal W. Fleming

**Affiliations:** Department of Anesthesiology & Pain Medicine, University of California, Davis, 4150 V Street Suite 1200 PSSB, Sacramento, CA 95817 USA

**Keywords:** Diastolic dysfunction, Mitral annular tissue velocity (e’), Intravascular volume

## Abstract

**Background:**

Abnormal diastolic function is an independent predictor of adverse postoperative outcomes. Mitral annular tissue Doppler velocity (e’) is a key parameter for assessing diastolic function. The purpose of this study was to confirm that an acute increase in preload did not significantly impact the intraoperative measurement of e’ and secondarily evaluate the impact of this acute intravascular volume increase on the clinical assessment of diastolic function using a previously described simplified algorithm.

**Methods:**

This was a prospective, non-randomized study in adult patients undergoing elective cardiac surgeries requiring transesophageal echocardiographic monitoring, arterial pressure and Swan-Ganz catheter placements as part of the surgical procedure. Following baseline echocardiographic and hemodynamic measurements, 500 ml of crystalloid solution was infused over 10 min. Hemodynamic and echocardiographic measurements were repeated 5 min after fluid administration.

**Results:**

Complete data sets were available from 84 of the 100 patients who were enrolled in this study. There was no significant change in the values of e’. The average baseline was 7.8 ± 2.0 cm/s (95%CI: 7.4, 8.2) and 8.1 ± 2.4 (95%CI: 7.6, 8.6) following the fluid bolus (*p* = 0.10). All hemodynamic variables associated with increased intravascular volume (central venous pressure, pulmonary arterial pressures and stroke volume variation) changed significantly. The overall distribution of diastolic function grades did not change following fluid administration (*p* = 0.69). However, there were many individual patient differences. When using this simplified algorithm, functional grading changed in 35 patients. Thirty of these 35 changes were only a single grade shift. 22 patients had worse functional grading after fluid administration while 13 had improved grading. Nine patients with normal diastolic function at baseline demonstrated diastolic dysfunction after fluid administration while 6 patients with baseline dysfunction normalized following the fluid bolus.

**Conclusion:**

We confirmed that e’ is a robust measurement that is reproducible in the intraoperative setting despite variable vascular volume loading conditions, however, the clinical assessment of diastolic function was still altered in 42% of the patients following an intravenous fluid bolus.

## Background

Diastolic dysfunction (DD) implies a left ventricular (LV) filling abnormality of reduced compliance and/or impaired relaxation. Abnormal diastolic function exists in more than 50% of patients presenting for cardiac or high-risk non-cardiac surgery and has been shown to be an independent predictor of adverse postoperative outcomes including prolonged ventilation, longer intensive care unit length of stay, and death in this patient population [1–3]. The American Society of Echocardiography (ASE) and European Association of Echocardiography (now European Association of Cardiovascular Imaging [EACVI]) released the first guidelines document for the diagnosis of DD in 2009 [4]. The extensive number of variables required to fit these original algorithms limited their clinical utility. The ASE and EACVI revised the algorithms in 2016, limiting the number of variables used to assess diastolic function to four key measurements: early diastolic tissue velocity (septal, and lateral e’), the average early trans-mitral filling velocity (E)/e’ ratio, left atrial volume indexed to body surface area, and tricuspid regurgitation velocity [5]. A simplified algorithm has been described by Swaminathan et al. with demonstrated utility in the intraoperative setting [6].

Given the management and prognostic implications of diastolic dysfunction, we sought to confirm the robustness of lateral wall mitral annular tissue velocity (e’) as one of the key parameters in the diagnostic algorithms in the often-dynamic intraoperative setting. Previous studies have examined the effects of drugs, patient position and intravenous fluids on mitral tissue Doppler measurements with mixed conclusions [7, 8]. This study was designed to specifically evaluate the effect of an acute increase in preload on e’. The primary aim was to confirm that an acute increase in preload would not significantly impact the measurement of e’. The secondary study aim was to evaluate the effect of an acute volume load on the assessment of diastolic function. We hypothesized that an acute volume load would not significantly change the measurement of e’ or the classification of diastolic function.

## Methods

This study was a data sub-set analysis from a University of California, Davis Human Subjects Research Committee-approved protocol that investigated the utility of the plethysmographic variability index (PVI) as a parameter to predict fluid responsiveness (https://clinicaltrials.gov/ Identifier: NTC03075150). All patients provided written informed consent prior to enrollment. The data reported here was collected as part of this single center, prospective, non-randomized, interventional study that recruited patients who were scheduled for elective surgery requiring general anesthesia and mechanical ventilation. Inclusion criteria included age greater than 18 years and arterial pressure and Swan-Ganz catheter placements indicated as part of the scheduled surgical procedure. Patients who presented with cardiac arrhythmias or intracardiac shunts were excluded. The study population was comprised of patients scheduled for elective cardiac surgical procedures.

No restrictions were placed on peri-operative anesthetic management. The majority of the patients received intravenous midazolam (1-2 mg) for premedication and radial arterial catheters were placed prior to arrival in the operating room (OR). After transfer to the OR table, standard monitors were placed and general anesthesia was induced with a combination of intravenous fentanyl and propofol. Rocuronium was administered to facilitate endotracheal intubation and anesthesia was maintained with a combination of additional fentanyl and sevoflurane as clinically indicated. Following induction of general anesthesia and endotracheal intubation, the pulmonary artery catheter was inserted via the internal jugular vein and transesophageal echo (TEE) probe was placed (Phillips Epic7C, X8-2t or X7-2t transducer, Philips Healthcare, Andover MD). Pulsed wave Doppler measurement of trans-mitral flow velocities were recorded along with tissue Doppler imaging (TDI) of the lateral mitral annular ring using a mid-esophageal 4-chamber or 2-chamber view. Syngo® Dynamics software (Siemens Medical Solutions, Malvern, PA) was used for image capture and measurements.

Patients were studied prior to sternotomy. After a 5-minute period of hemodynamic stability with no changes in anesthetic management, sevoflurane concentration, vasopressor administration and no increased intravenous fluid administration, baseline hemodynamic measurements, including cardiac output, systemic arterial, pulmonary arterial and central venous pressures were recorded along with an echocardiographic assessment of left ventricular diastolic function. For the mitral flow velocities, TEE was used to determine peak early mitral flow velocity (E), peak late mitral flow velocity (A) and lateral wall mitral annular TDI velocity (e’), with E/A and E/e’ ratios calculated subsequently. Patients then received an intravenous fluid bolus consisting of 500 cc of crystalloid solution infused over 10 min. A second set of hemodynamic and TEE measurements were collected 5 min after completion of this fluid administration.

### Data analysis

**Fig. 1:**
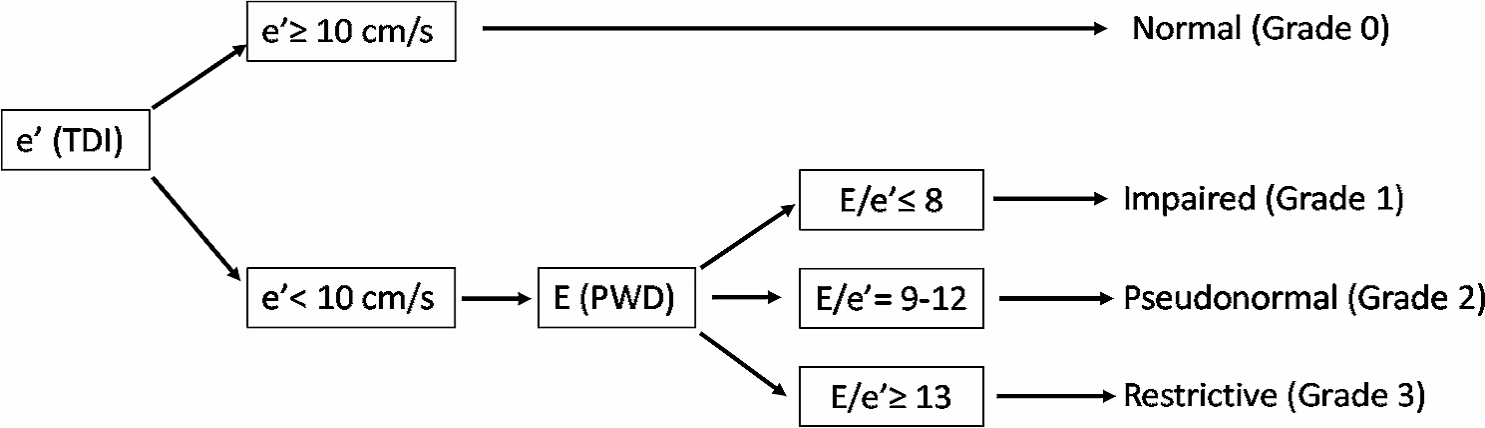
Assessment of diastolic function. A simplified algorithm for the assessment of diastolic function adapted from Swaminathan et al. [6] e’ – lateral mitral annular early diastolic tissue velocity. TDI – tissue Doppler imaging. E – Early trans-mitral filling velocity (cm/s). PWD – pulse wave Doppler

Diastolic function was graded for all patients before and after the intravenous fluid bolus with the echocardiographic measurements using a simplified version of the algorithm described by Swaminathan et al. [6] (Fig. 1), based on tissue doppler velocity (e’) and the trans-mitral E/e’ ratio. Grades included: grade 0 (normal function), grade 1 (impaired relaxation), grade 2 (pseudonormal) and grade 3 (restrictive). Diastolic dysfunction was present if e’ was < 10 cm/s. Subsequent grading was determined by evaluating the ratio of trans-mitral early wave to the lateral mitral annular early diastolic tissue velocity (E/e’). Values less than or equal to 8 indicated grade 1, or impaired relaxation, E/e’ values between 9 and 12 defined grade 2 or pseudonormal, and values greater than or equal to 13 were categorized as restrictive. For this classification, E/e’ values were rounded to the nearest whole number.

### Statistical analysis

Statistical comparisons were made using Graph-Pad Prism (Version 10.1.2 for Windows, Graph-Pad Software Inc, San Diego, CA). The D’Agostino & Pearson test was used to assess normality of each data set. The distributions of all measured left ventricular (LV) functional parameters and ratios were evaluated for normality. Comparisons of measured parameters before and after volume expansion were done using a paired two-tailed t-test for normally distributed data. The Wilcoxon matched-pairs signed rank test was used for non-normally distributed data. Comparisons of unpaired data among groups were done using Tukey’s multiple comparison test for normally distributed data and Dunn’s multiple comparison test for non-normally distributed data. Comparisons of ordinal data utilized Chi-square analysis. Results are expressed as mean +/- standard deviation for variables with a normal distribution and median [25%-75% IQR] for variables with a non-normal distribution. A value of p < 0.05 was considered to be statistically significant. Power analysis was performed retrospectively with the following parameters: α = .05, a known population average of lateral wall e’: 9.6 ± 2.8 cm/s (60≥ age) [6] and a pre-bolus average from this study of 7.8 cm/s.

## Results

Between December, 2016 and April, 2018, a total of 100 consecutive patients meeting the inclusion criteria were considered eligible and enrolled in this study. After review of all echocardiographic measurements, 84 patients contained complete, accurate and verifiable data measurements. 13 patients were not included because complete raw data was missing, specifically e’ or E measurements. Two patients were excluded from the final analysis due to poor quality waveforms and one patient was excluded due to a recording error. Given the study design, power analysis could only be performed retrospectively. For a power of 0.95 and α = 0.05, the estimated sample size was *n* = 31, confirming this analysis was adequately powered for the primary variable.

Demographic and clinical characteristics are summarized in Table 1. The patients in this study could be categorized into four groups: elective valvular replacement or repair, with or without coronary artery disease. Patients had a spectrum of disease pathology, including heart failure, isolated valvular pathology and valvular pathology with coronary artery disease necessitating coronary artery bypass grafting (CABG). None of the patients in this study group had severe mitral stenosis, significant mitral annular calcification or lateral wall motion abnormalities. Data was collected from 27 females and 57 males. Patients had a median age of 66 [60, 73] years and median BMI of 28 [25,32] kg·m^-2^. The majority (73) were American Society of Anesthesiology (ASA) Physical Status (PS) 4. Ten patients were ASA PS 3 and one was ASA PS 2.

**Table 1:** Demographic and clinical characteristics

Age (years)	65±11.1
BMI	29 ± 5.2
ASA Class (2/3/4)	(1/10/73)
Valvular Pathology	
Aortic Stenosis	
Mild/Moderate	11(13%)
Severe	16(19%)
Aortic Regurgitation	
Mild/Moderate	21(25%)
Severe	5(6%)
Mitral Stenosis	
Mild/Moderate	7(8%)
Mitral Regurgitation	
Mild/Moderate	27 (32%)
Severe	7(8%)
Heart Failure	
HFrEF (< 50%)	9 (12%)
Coronary Artery Disease	39(46%)

BMI – Body Mass Index, ASA – American Society of Anesthesiologists, HFrEF – Heart Failure with reduced Ejection Fraction

Static pressures and dynamic hemodynamic variables recorded before and after the fluid bolus are summarized in Table 2. The distribution of these measurements was evaluated for normality. Only the cardiac output (CO) data was normally distributed so the Wilcoxson matched-pairs rank sign test was used for all paired comparisons. All of the measured parameters, except CO, demonstrated statistically significant changes. However, only the parameters associated with the increase in preload demonstrated changes that could be considered clinically significant. The central venous pressures (CVP) increased by 47% from 7.0[5.0, 9.0] to 10 [ 7.0,12.0] mmHg following the fluid bolus. The pulmonary arterial pressures (PAP) increased by 10%, 26% and 17% for systolic (24 [12,31] to 26 [22,34] mmHg), diastolic (11 [9,14] to 14 [11,17] mmHg) and mean (17 [14,19] to 19 [16,23] mmHg) pressures respectively. The stroke volume variation (SVV) decreased by 24% from 8 [7,11] to 6 [4,8].

**Table 2:** Hemodynamic measurements

Parameter	Before	After	p
**CVP (mmHg)**			
mean ± SD	7.4 ± 3.3	10.0 ± 3.8	< 0.0001
Median [25-75% IQR]	7.0[5.0,7.0]	10 [ 7.0,12.0]	
**SPAP (mmHg)**			
mean ± SD	26 ± 7.5	28 ± 8.0	< 0.0001
Median [25-75% IQR]	24 [12,31]	26 [22,34]	
**DPAP (mmHg)**			
mean ± SD	12 ± 5.0	15 ± 6.0	< 0.0001
Median [25-75% IQR]	11 [9,14]	14 [11,17]	
**MPAP (mmHg)**			
mean ± SD	18 ± 6.1	21 ± 6.8	< 0.0001
Median [25-75% IQR]	17 [14,19]	19 [16,23]	
**SVV (%)**			
mean ± SD	8.9 ± 3.3	6.7 ± 3.6	< 0.0001
Median [25-75% IQR]	8 [7,11]	6 [4,8]	
**CO (L/min)**			
mean ± SD	4.0 ± 1.1	4.1 ± 1.1	= 0.18
Median [25-75% IQR]	4.1[3.3,4.8]	4.0[3.4,4.9]	
**HR (bpm)**			
mean ± SD	68 ± 13	65 ± 12	< 0.0001
Median [25-75% IQR]	66[59,77]	63[56,72]	
**SAP (mmHg)**			
mean ± SD	122 ± 20	110 ± 15	< 0.0001
Median [25-75% IQR]	118[108,137]	110[98,121]	
**DAP (mmHg)**			
mean ± SD	60 ± 11	56 ± 9	< 0.0001
Median [25-75% IQR]	60[53,66]	56[50,61]	
**MAP (mmHg)**			
mean ± SD	80 ± 13	74 ± 11	< 0.0001
Median [25-75% IQR]	80[72,89]	73[66,83]	

CVP – Central Venous Pressure, SPAP – Systolic Pulmonary Artery Pressure, DPAP – Diastolic Pulmonary Artery Pressure, MPAP – Mean Pulmonary Artery Pressure, SVV – Stroke Volume Variation, CO – Cardiac Output, HR – Heart Rate, SAP – Systolic Arterial Pressure, DAP – Diastolic Arterial Pressure, MAP – Mean Arterial Pressure

**Fig. 2:**
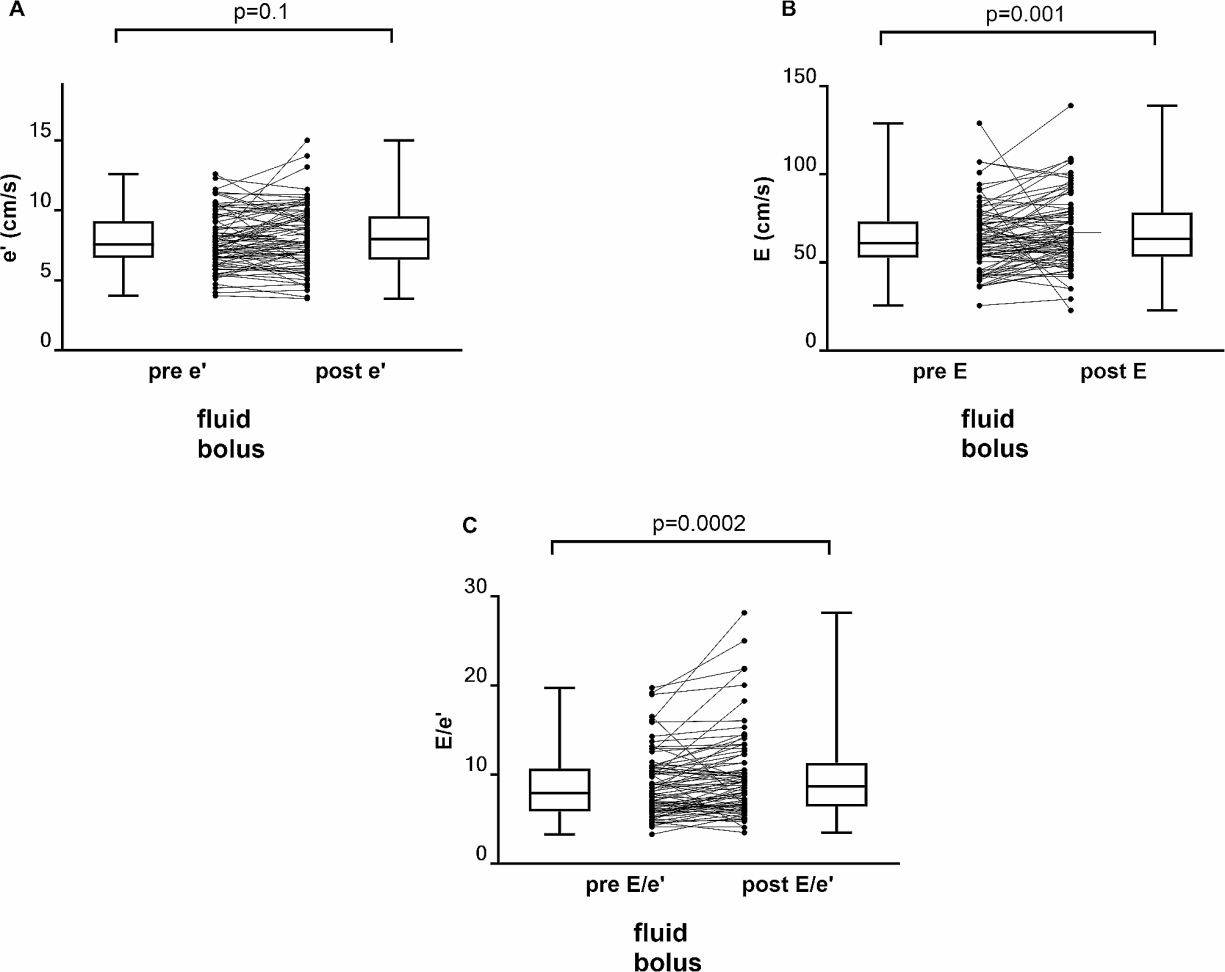
(**A**) Box plot (median, 25%-75% inter-quartile range, whiskers minimum/maximum) and individual patient values of e’ (lateral mitral annular early diastolic tissue velocity) before and after the administration of an intravenous fluid bolus (500 ml). (**B**) Box plot (median, 25-75% inter-quartile range, whiskers minimum/maximum) and individual patient values of E (early trans-mitral blood flow velocity) before and after the administration of an intravenous fluid bolus (500 ml). (**C**) Box plot (median, 25-75% inter-quartile range, whiskers minimum/maximum) and individual patient values of E/e’ ratio before and after the administration of an intravenous fluid bolus (500 ml)

Primary echocardiographic data included lateral mitral annular velocity (e’), peak early ventricular diastolic filling velocity (E) and late filling velocity (A). The distributions of these measured LV functional parameters were evaluated for normality. Only the baseline values of e’ passed the normality test so the Wilcoxson matched-pairs rank sign test was again used for all paired comparisons. The average baseline e’ was 7.8 ± 2.0 cm/s (7.6 [6.6,9.2]) and 8.1 ± 2.4 (7.9 [6.5, 9.6]) following the fluid bolus (*p* = 0.10). In contrast, E increased from a baseline value of 63 ± 18.4 cm/s (61 [53,73]) to 66 ± 20.5 (63 [53,78]) following the fluid bolus (*p* = 0.001). Consequently, the E/e’ ratio increase from a baseline value of 8.7 ± 3.6 (7.9 [5.9,11]) to 9.6 ± 4.7 (8.7 [6.4,11]) was also statistically significant (*p* = 0.0002) (Fig. 2). The average baseline late filling velocity (A) was 59 ± 21.9 cm/s (56 [43.70]) and 61 ± 21.3 (59 [41,79]) following the fluid bolus (*p* = 0.69) (Table 3).

**Table 3:** Echocardiographic measurements

Parameter	Overall		P
	Before	After	
**e’ (cm/s)**			
mean ± SD	7.8 ± 2.0	8.1 ± 2.4	0.10
Median [25-75% IQR]	7.6 [6.6,9.2]	7.9 [6.5, 9.6]	
**E (cm/s)**			
mean ± SD	63 ± 18	66 ± 20*	0.001
Median [25-75% IQR]	61 [53,73]	63 [53,78]	
**E/e’**			
mean ± SD	8.7 ± 3.6	9.6 ± 4.7*	0.0002
Median [25-75% IQR]	7.9 [5.9,11]	8.7 [6.4,11]	
**A (cm/s)**			
mean ± SD	59 ± 21.9	61 ± 21.3	0.69
Median [25-75% IQR]	56 [43.70]	59 [41,79]	

e’ – Mitral annular tissue Doppler velocity, E – Early peak trans-mitral flow velocity, A – Late peak trans-mitral flow velocity

**Fig. 3:**
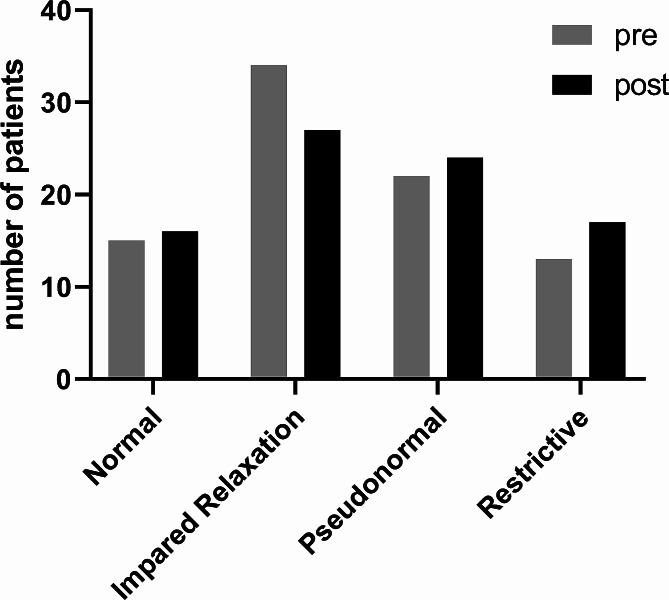
Distribution of diastolic function grades before (pre) and after (post) an intravenous fluid bolus of 500 ml

All patients were categorized before and after the fluid bolus by their diastolic function grades based on the Swaminathan algorithm [6]. The overall distribution of diastolic function grades did not change following fluid administration (chi square analysis; χ^2^ = 1.45, df = 3, *p* = 0.69) (Fig. 3).

**Fig. 4:**
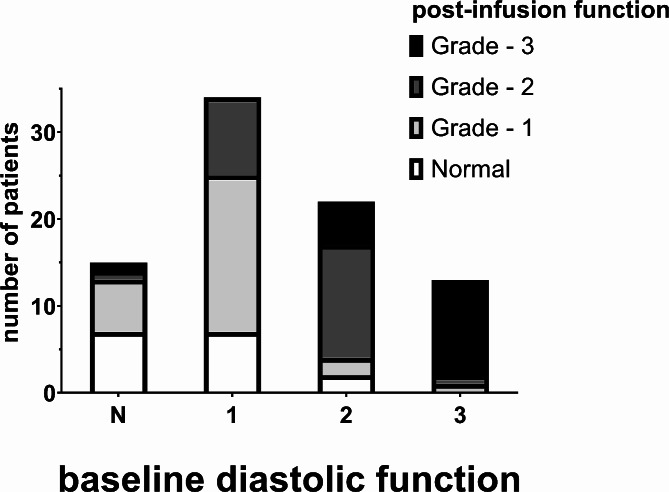
Summary of individual patient changes in diastolic function assessment. Baseline assessments are represented by the total column height. Assessments after a volume infusion are summarized by the shading within each column

However, there were many individual patient differences. Of the 35 patients (42%) with changes, 22 patients had worsening functional grading, 20 by one grade, 1 by two grades and 1 by three grades. 11 patients who were normal or grade 1 shifted to grade 2 or grade 3. When compared to patients whose functional assessment did not change, these patients had a greater baseline e’ (8.4[7.4,10] vs. 7.1[6.0,8.1], *p* = 0.009) but comparable baseline E/e’ (7.7[5.9,9.8 vs. 8.1[6.0,11], *p* > 0.99). All demographic and baseline hemodynamic variables were also comparable between these two groups. The change in e’ was greater in this group of patients (-0.7[-2.2,-0.03] vs. 0.3[-0.2,0.9], *p* = 0.003) and the change in E/e’ was statistically greater (2.1[1.0,3.7] vs. 0.6[-0.5,1.6], *p* = 0.003). The changes in all hemodynamic variables were comparable between these two groups. 13 patients had improved functional grading, 10 by one grade and 3 by two grades. 5 patients who were grade 2 or grade 3 shifted to normal or grade 1. When compared to patients whose functional assessment did not change, these patients had a comparable baseline e’ (7.9[6.5,9.5] vs. 7.1[6.0,8.1]), *p* = 0.44) and comparable baseline E/e’ (7.5[5.2,10] vs. 8.1[6.0,11], *p* > 0.99). Demographic and baseline hemodynamic variables were also comparable between these two groups, with the exceptions of age (56 ± 14 vs. 67 ± 11, *p* = 0.04), diastolic arterial pressure (67 ± 7.4 vs. 59 ± 13, *p* = 0.02 and heart rate (79 ± 15 vs. 66 ± 12, *p* = 0.01). The change in e’ was greater in this group of patients (1.7[0.9,2.7] vs. 0.3[-0.2,0.9], *p* = 0.02) and the corresponding decrease in E/e’ was also greater (-0.8[-2.7,0.4] vs. 0.6[-0.5,1.6], *p* = 0.03). The changes in all hemodynamic variables were comparable between these two groups, with the exception of heart rate (-12 ± 12 vs. -2.3 ± 9.7, *p* = 0.04). These individual patient grade changes are summarized in Fig. 4.

The E/e’ values were analyzed to further evaluate the grade assessment shifts between normal/grade 1 and grade 2/grade 3, There was no correlation between baseline vales of E/e’ and the change after fluid administration (Pearson *r* = 0.02 [-0.19, 0.24], *p* = 0.8). However, 11 of the 53 patients with a baseline value of E/e’<9 increased to > 9 after fluid administration and the majority (8) of these patients had baseline vales between 7.5 and 9. Only 2 of the 31 patients with baseline values of > 9 decrease to < 9.

## Discussion

Major risk factors for diastolic dysfunction (DD) include increasing age, hypertension, diabetes mellitus, and left ventricular hypertrophy. Epidemiological evidence suggests that there is a latent phase in which diastolic dysfunction is present and progresses in severity before symptoms can be appreciated [9]. DD is increasingly prevalent in the surgical patient population. Asymptomatic, mild left ventricular diastolic dysfunction (LVDD) is present in 21% and moderate or severe DD is present in 7% of the population [10]. Up to 34% of patients with diabetes mellitus have DD [11].

Diastolic dysfunction is of clinical significance given the increased risk for prolonged mechanical ventilation, ICU readmission, hospital length of stay, or death in patients undergoing aortic valve repair and/or CABG [3]. DD in hospitalized patients has been shown to be a predictor of all cause morbidity and mortality [12, 13]. When diagnosed perioperatively, DD has had a significant association with postoperative CHF and post-operative length of stay after vascular surgery [1]. Further supporting evidence that the echocardiographic measurements we surveyed have clinical utility include studies that demonstrated patients with lower e’ and higher E/e’ ratio have an increased mortality when presenting with severe sepsis or shock [14, 15] and an increased incidence of failure to wean from mechanical ventilation [16].

Despite the growing incidence and clinical implications, pre-operative assessment of diastolic function is not consistently available. Assessment of diastolic function is not a part of the standard comprehensive intraoperative echocardiographic examination [1]. This may reflect the absence of therapeutic options when diagnosing DD or the lack of an assessment algorithm that is both practical and has clinical utility. However, given the clinical implications for care in the post-operative period, there is a need to identify DD to prognosticate, and potentially improve post-operative outcomes.

In this prospective study, we confirmed that e’ is a robust measurement that is reproducible in the intraoperative setting despite the variable loading conditions that occur in patients undergoing general anesthesia with positive pressure ventilation. When assessing the preload status in this study, the central venous pressure, pulmonary arterial pressures, as well as mean arterial pressure all significantly increased following the fluid bolus, but e’ did not significantly change. This is consistent with previous studies that show e’ is a preload-independent estimation of LV filling pressure, unlike mitral inflow variables that are load dependent because they reflect the pressure difference between the left atrium and ventricle during diastole [5, 14, 17]. However, there are assessments in some clinical settings that suggest e’ can be modified by changes in preload [7, 8].

The absence of a statistically significant impact of acute fluid administration on e’ measurement parallels the absence of any significant change in the overall distribution of diastolic function grades in the study population. However, small changes in e’, in combination with the expected changes in trans-mitral flow velocities did result in changes of the diastolic function grading in a significant number of individual patients. These effects were variable but were observed in slightly under half (42%) of the patients in this study. Within this group, approximately 2/3 of the patients had a worsening of their diastolic functional grading while 1/3 had an improvement. Although many of the observed changes in functional grading are not of clinical significance, nine patients with normal diastolic function developed dysfunction following the fluid bolus while six patients with DD before the fluid bolus normalized after the fluid administration. There were no clear distinctions with respect to baseline echocardiographic or hemodynamic characteristics of these patients in whom functional grading changed and no distinguishing changes following fluid administration. The prognostic implications of such changes are not clear as they seem to largely reflect minor variations in measurements around the fixed numerical values used for classification. Caution should always be used when considering the implications associated with the conversion of a continuous variable (e’) to a categorical variable (diastolic functional grade).

Some limitations to these observations should be highlighted. We used a simplified algorithm for assessing diastolic function which has been validated in the clinical setting [6]. However, a more comprehensive algorithm may have provided different assessments. The intraoperative hemodynamic state varies for many reasons. Our measurements looked specifically at acute volume changes and are only a “snapshot” of what is both a continuous and dynamic process. Additional influences include the intravenous and inhalational drugs used for induction and maintenance of anesthesia. In patients without cardiac pathology, propofol has been shown to worsen diastolic function [18] while actually improving function in patients with existing diastolic dysfunction [15]. Inhalational anesthetics such as sevoflurane and isoflurane have been shown to improve diastolic function [19]. This protocol did not specifically control for the impact of these anesthetic agents. In addition, this study was not large enough to control for pathologies that could impact the echocardiographic assessments. Patients with pathologies known to impact the measurement of e’ such as severe annular calcification or mitral stenosis were not included, however, early diastolic filling velocities decrease with age and mitral regurgitation results in an E-dominant trace. Also, in patients with mitral stenosis measurements may reflect flow across the stenosis rather than intrinsic diastolic dyfunction [17]. Lastly, the subsets of patients with changes in functional assessments were not large enough to allow characterization of any echocardiographic or hemodynamic characteristics that might be associated with the observed changes.

## Conclusions

This study provides guidance regarding assessment of diastolic function in the intra-operative setting. For these patients, e’ was a robust parameter that was pre-load independent in the setting of an acute intravascular volume increase. However, individual patient assessments of diastolic function may still change. Intra-operative measurement of e’ may be a reasonable guide for both management and prediction of post-operative outcomes. Further studies are warranted to assess how this value may change throughout the perioperative period and to evaluate the clinical care implications of changes in diastolic functional grading.

## Data Availability

The datasets used and/or analyzed during the current study are available from the corresponding author on reasonable request.

## Abbreviations

A: Peak Late Mitral Flow Velocity
ASA: American Society of Anesthesiologists
ASE: American Society of Echocardiography
BMI: Body Mass Index
CABG: Coronary Artery Bypass Grafting
CO: Cardiac Output
CVP: Central Venous Pressure
DAP: Diastolic Arterial Pressure
DPAP: Diastolic Pulmonary Artery Pressure
DD: Diastolic Dysfunction
e’: Mitral annular tissue Doppler velocity
E: Peak Early Mitral Flow Velocity
EACVI: European Association of Cardiovascular Imaging
HFrEF: Heart Failure with reduced Ejection Fraction
HR: Heart Rate
LV: Left Ventricle
MAP: Mean Arterial Pressure
MPAP: Mean Pulmonary Artery Pressure
OR: Operating Room
PVI: Plethysmographic Variability Index
SAP: Systolic Arterial Pressure
SPAP: Systolic Pulmonary Artery Pressure
SVV: Stroke Volume Variation
TDI: Tissue Doppler Imaging
TEE: Transesophageal Echo
